# Estimation of the Lateral Ventricles Volumes from a 2D Image and Its Relationship with Cerebrospinal Fluid Flow

**DOI:** 10.1155/2013/215989

**Published:** 2013-09-16

**Authors:** Chaarani Bader, Capel Cyrille, Zmudka Jadwiga, Daouk Joel, Anthony Fichten, Gondry-Jouet Catherine, Bouzerar Roger, Balédent Olivier

**Affiliations:** ^1^Department of Imaging, Jules Verne University of Picardy and Amiens University Hospital, 80054 Amiens Cedex, France; ^2^Department of Neurosurgery, Jules Verne University of Picardy and Amiens University Hospital, 80054 Amiens Cedex, France; ^3^Department of Geriatrics, Jules Verne University of Picardy and Amiens University Hospital, 80054 Amiens Cedex, France; ^4^Department of Radiology, Jules Verne University of Picardy and Amiens University Hospital, 80054 Amiens Cedex, France

## Abstract

*Purpose*. This work suggests a fast estimation method of the lateral ventricles volume from a 2D image and then determines if this volume is correlated with the cerebrospinal fluid flow at the aqueductal and cerebral levels in neurodegenerative diseases. *Materials and Methods*. FForty-five elderly patients suffering from Alzheimer's disease (19), normal pressure hydrocephalus (13), and vascular dementia (13) were involved and underwent anatomical and phase contrast MRI scans. Lateral ventricles and stroke volumes were assessed on anatomical and phase contrast scans, respectively. A common reference plane was used to calculate the lateral ventricles' area on 2D images. *Results*. The largest volumes were observed in hydrocephalus patients. The linear regression between volumes and areas was computed, and a strong positive correlation was detected (*R* ^2^ = 0.9). A derived equation was determined to represent the volumes for any given area. On the other hand, no significant correlations were detected between ventricles and stroke volumes (*R* ^2^ ≤ 0.15). *Conclusion*. Lateral ventricles volumes are significantly proportional to the 2D reference section area and could be used for patients' follow-up even if 3D images are unavailable. The cerebrospinal fluid fluctuations in brain disorders may depend on many physiological parameters other than the ventricular morphology.

## 1. Introduction

It is still not well elucidated if cerebrospinal fluid (CSF) oscillations amplitude can be associated with ventricle dilatations and how its volume variations are implicated in brain disorders.

The most common forms of brain disorders in the elderly regroup Alzheimer's disease (AD), vascular dementia (VaD), and also normal pressure hydrocephalus (NPH). 

Alzheimer disease is the leading cause of dementia and is characterized by a progressive decline in cognitive function, which typically begins with deterioration in memory. Recent epidemiological studies predict a doubling of its global prevalence every 20 years, estimated to be actually as high as 24 million affected persons in the world [1].

VaD includes all forms of dementia related to cerebrovascular diseases. Its prevalence ranges from 5 to 31 per 1000 and may vary depending on the studied population [2]. Studies from various countries found that the proportion of dementia cases diagnosed as vascular dementia ranged from 10 to 38% making it the second most common cause of dementia after Alzheimer's disease [2].

The number of people who develop hydrocephalus or who are currently living with it is difficult to establish since there is no national registry or database of affected people. However, recent population-based studies have estimated the prevalence of NPH to be about 0.5% in those over 65 years old, with an incidence of about 5.5 patients per 100,000 of people per year [3, 4]. 

NPH is a curable disease associated with ventricular dilation and the presence of a part or total symptoms of Hakim's triad [5] such as cognitive impairment, gait disturbance, or urinary problems. Nevertheless, it is not a pathology with normal intracranial pressure (ICP) according to Bret et al. [6]. During overnight ICP monitoring, many B-waves can be observed, and during infusion study with ICP monitoring, pressure volume compensatory reserve parameters are altered [5].

These three brain disorders (AD, VaD, and NPH) are distinguished by their specific pathological features. In contrast, one of the common features revealed by MR neuroimaging is the dilatation of the lateral ventricular system, which is more severe in the group with NPH. Nevertheless, difficulties still exist in the differential diagnosis between atrophy, AD, NPH, or VaD in a nonnegligible part of elderly patients presenting moderate ventricular dilation associated with moderate Hakim triad symptoms. Recent studies have shown that CSF flow investigation at the aqueductal and cervical levels could help also to differentiate between these brain disorders, particularly between AD and NPH [7]. However, it remains unclear whether this can be related to altered ventricular volumes or to other pathological aspects. 

Chiang et al. [8] detected a strong linear correlation of the CSF displaced through the aqueduct during cardiac cycle with the ventricular volume, notably with the total ventricular volume (*r* = 0.838), where measurements of the ventricular system were manually made by tracing the ventricle's boundaries on 3D anatomical T1-weighted MR images. 

Measurements of the lateral ventricles are usually made either by using computer-assisted manual tracing methods and semiautomatic algorithms or fully automatic ones. Manual tracing methods are commonly applied on 3D MR images on each slice of the series. This process may be very time consuming when the amount of data is huge since, and unlike the 2D sequences, the 3D images acquisition and the following post-processing are two operations that need time and thoroughness. Automatic segmentation algorithms usually require a 3D MR image for the registration step, which may need a considerable time too, even on powerful machines. 3D MR long acquisitions depend on the subject's condition and are very motion sensitive, and in consequence, this 3D approach is limited in clinical practice when subjects are patients with neurodegenerative diseases who cannot remain in a stable condition during a long acquisition and who often present movement and shaking troubles. This leads to artifacted 3D images that are difficult to process since it may contain false information. Moreover, the postsegmentation algorithms of 3D images use anatomical brain templates as a reference for the registration step, averaged from brain images of young and healthy subjects [9]. These templates are not compatible with elderly patients with neurodegenerative disease. 

A previous study [10] tried to overcome this problem by using 2D indexes to evaluate the ventricular morphology variations in NPH patients through single 2D slices on computed tomography. They observed that the size of the ventricular system calculated using scan indexes such as the ventricular brain ratio, bicaudate, bifrontal ratios, and the third ventricle-Sylvian fissure ratio may correlate with the severity of the symptoms in patients with hydrocephalus. However, this approach was not evaluated with other brain disorders. 

In this work, we suggested, following the same concept of the 2D bicaudal and bifrontal indexes, the hypothesis that a single 2D slice through the ventricles could represent an acceptable approximation of the total ventricle volume. We evaluated this 2D approach by comparing quick ventricle area measurements with the total ventricle volumes in a large neurodegenerative population presenting small, normal, large, and extra-large ventricles in patients with AD, NPH, and VaD. In addition, this study aims to determine if a relationship exists between the CSF ventricular volume and CSF pulsatilities in patients with such brain disorders.

## 2. Materials and Methods

### 2.1. Subjects

A total of 45 elderly patients participated in this study. Subjects were consecutively selected from among patients who visited the university hospital starting from 2009 to 2011 and agreed to sign an inform consent to participate in the research. Patients were divided into 3 groups (AD, NPH, and VaD) after a comprehensive physical and neurological examinations performed by an experienced neurologist; it included a screen of cognitive functions and disability in activities of daily living using the IADL questionnaire. AD and VaD patients belong to an ongoing clinical study (NCT01815112) and were diagnosed according to the NINCDS-ADRDA and the NINDS-AIREN criteria, respectively. 

The NPH group consisted of age-matched patients presenting 2 or 3 of the Hakim triad symptoms (gait troubles were mandatory) with a communicating aqueduct and and no macroscopic signs of CSF flow obstruction. In addition, patients had no antecedent events such as head trauma, intracerebral haemorrhage, meningitis or other known causes of secondary hydrocephalus. Based on these criteria and published guidelines [11], patients were classified as having “possible” NPH, and the diagnosis was confirmed by a neurosurgical consultation. NPH patients belong to a previous clinical study (NCT01815775).

Hence, this study involved 19 AD (age = 76 ± 4 years), 13 possible NPH (age = 71 ± 5 years), and 13 VaD patients (age = 73 ± 6 years) and was approved by the institutional review board.

### 2.2. MR Image Acquisition

All patients underwent the same brain examination with a standardized imaging protocol on a 3T MRI machine (General Electric Medical system, Milwaukee, WI). MR scans included 2D axial T2-weighted fluid-attenuated inversion recovery (FLAIR), axial T2* weighted, sagittal T1-weighted, 3D BRAVO (BRAinVOlume), and coronal T1-weighted anatomical imaging (sequence parameters detailed in Table 1). These MR data were used for segmentation and quantification of the lateral ventricles volumes and areas.

In addition to anatomic MRI scans, NPH, VaD, and 12 of the AD patients had phase-contrast MR imaging (PC-MRI) sequences for CSF flow added to the conventional clinical brain MR imaging protocol. Flow images were acquired with a fast 2D cine PC-MRI pulse sequence with k-space segmentation (with two views per segment) and a retrospective peripheral gating so that the 32 analysed frames covered the entire cardiac cycle (CC). 

**Figure 1:**
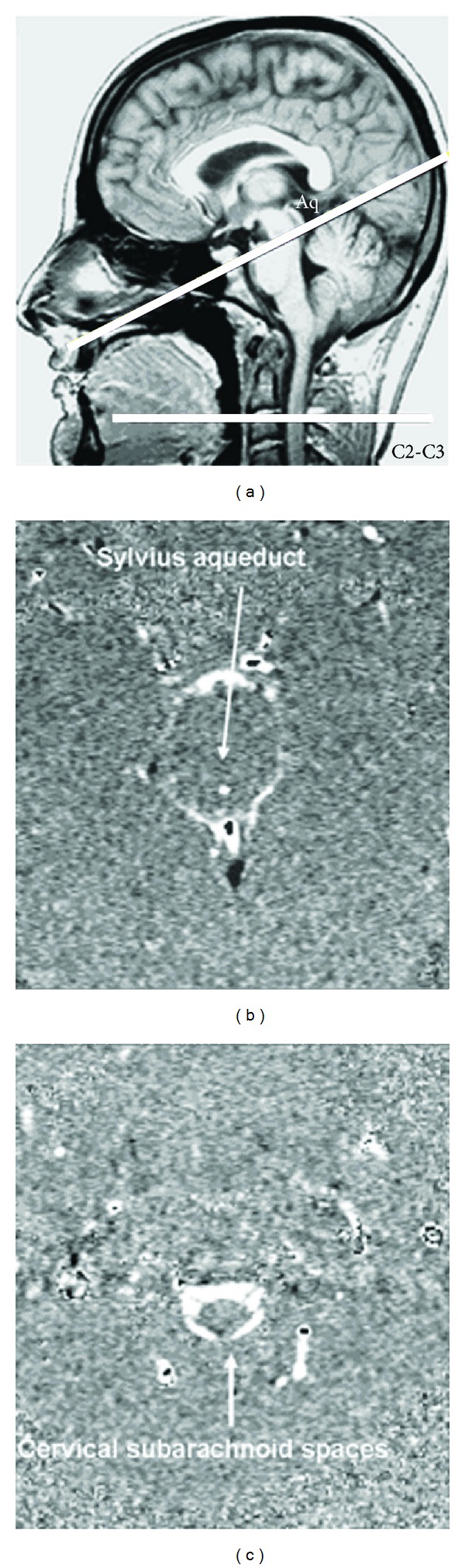
Data acquisition by phase-contrast magnetic resonance imaging: the selected acquisition planes were perpendicular to the presumed flow direction. Sections through the aqueduct of Sylvius and the C2-C3 subarachnoid space (C2-C3) were used for cerebrospinal fluid flow measurement (a). Examples of phase contrast images at the Sylvian aqueduct and the cervical subarachnoid spaces are shown in images (b) and (c).

Sagittal scout view sequences were used as localizers to select the aqueductal and cervical subarachnoidal spaces for CSF flow quantification level. The acquisition planes were selected perpendicular to the presumed direction of the flow and are represented in Figure 1. The acquisition time for each flow series was approximately 1 minute, with slight fluctuation that depended on the participant's heart rate. PC-MRI parameters are also detailed in Table 1. Velocity (encoding) sensitization was set to 10 or 20 cm/s for the aqueduct and 5 cm/s for the cervical subarachnoidal spaces. 

### 2.3. Image Processing and Analysis

For each patient in the 3 groups, we calculated the lateral ventricles volume (LvV), the lateral ventricles area (LvA), the aqueductal stroke volume (ASV), and the cervical stroke volume (CSV) values using the following image processing methods.

#### 2.3.1. Calculation of Lateral Ventricles Volume

**Figure 2:**
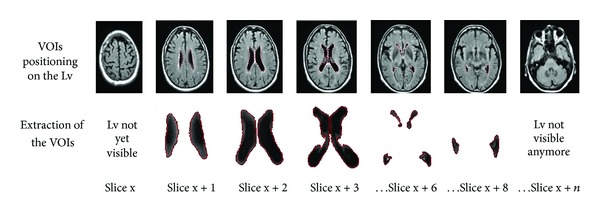
Semiautomatic segmentation with MIPAV applied for volume calculation of the Lv: In this example, VOIs were defined with a level set contour tool on the Lv of some slices of the 2D FLAIR sequence, then extracted in order to calculate the total volume. Lateral ventricles (Lv). Volumes of interest (VOIs).

LvV for each patient was assessed by tracing volumes of interest (VOIs) on each slice of the 3D images where the lateral ventricles were recognized (Figure 2).

VOIs' contours were defined semiautomatically with the level set VOI tool of MIPAV (http://mipav.cit.nih.gov/). In this method, MIPAV first analyzes the intensity values and uses the results from its Levelset algorithm to determine the probable boundary of the structure, and then it generates a contour. 

**Figure 3:**
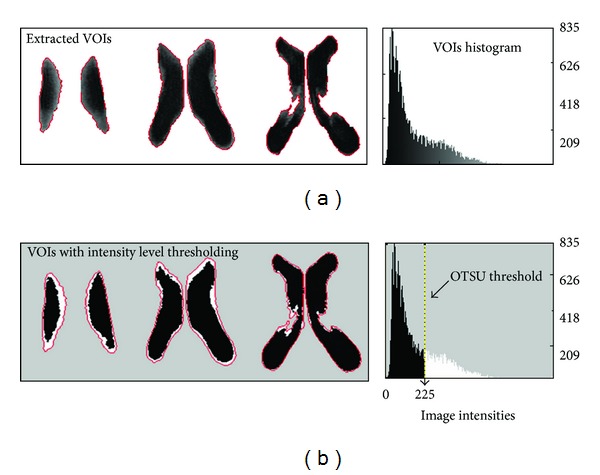
After the extraction of the VOIs by the level set algorithm (a), the partial volume effect on the boundaries of the Lv is isolated with an intensity threshold-based segmentation using the OTSU method (b) to calculate the optimal threshold on the histogram. This threshold is used to separate the gray level intensities into two clusters to form a binary image. The figure shows the effect of the intensity thresholding with a threshold value of 225, where the retained Lv voxels were colored in black. Lateral ventricles (Lv). Volumes of interest (VOIs).

These structures are mainly made of CSF, which may appear contrasted on T1 and T2-weighted MR acquisitions, but they also are prone to partial volume effects. This explains the nonuniform distribution of the voxel intensities inside the extracted VOIs observed on the histogram and may reduce the accuracy of the level set algorithm when determining the ventricle boundaries (Figure 3). In order to overcome this problem, the gray-level histogram was used to automatically set an optimal intensity threshold with the OTSU method [12] to separate the ventricles from the surrounding voxels, based on the slight contrast at their boundaries. The OTSU threshold-based segmentation method applied on the extracted VOIs assumes that each VOI contains two clusters of pixels, and then an optimal threshold separating those two clusters is calculated so that their intraclass variance is minimal. The calculated threshold by the OTSU method (Figure 3) was visually approved later by an experienced clinician.

The total volume was obtained by automatically counting the number of voxels within the segmented regions and then multiplying the number by the voxel size, including the intersection gap. Time needed to calculate the volume for one patient was on average 15 minutes on the 3D. An averaged lateral ventricles volume (LvV) was calculated as the mean of the volumes on the 3D sequences.

#### 2.3.2. Calculation of Lateral Ventricles Area

**Figure 4:**
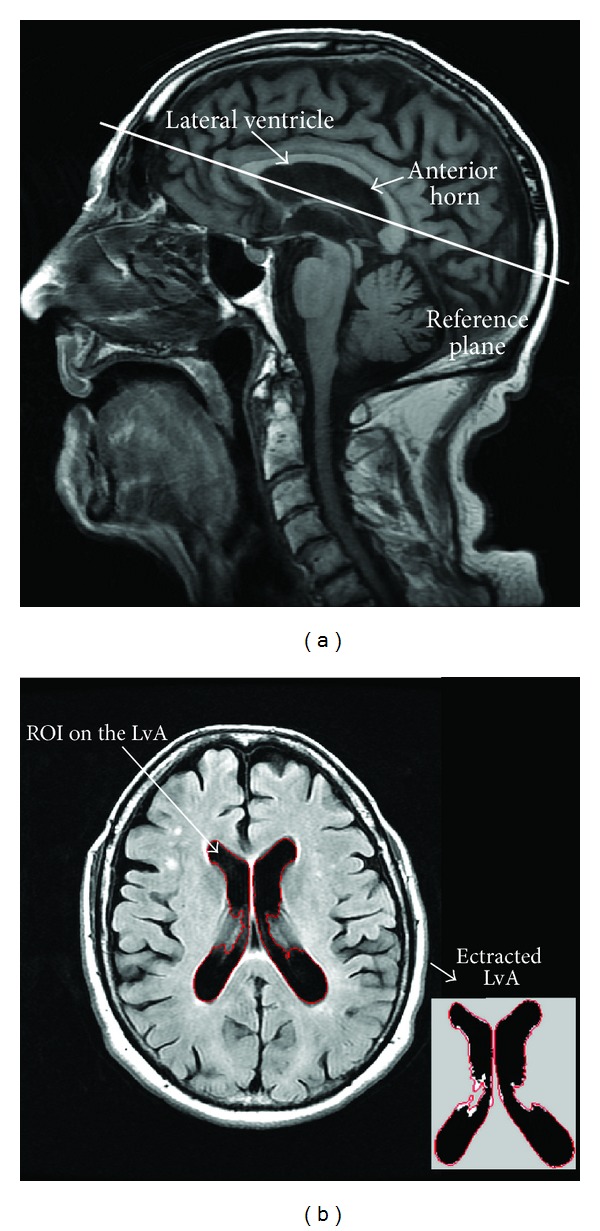
(a) Sagittal T1-weighted image showing the reference plane intersecting the anterior horn and the body of the lateral ventricles. (b) Segmentation of the lateral ventricles on the corresponding 2D FLAIR slice using the level set algorithm then thresholding to draw a region of interest and extract the lateral ventricles area.

A common reference plane intersecting the anterior horn and the body of the lateral ventricles was defined on the 2D sagittal images (Figure 4) then used on the 2D Flair and T2* axial images of all patients to calculate the lateral ventricles' surface area by applying the same segmentation method used to calculate volumes (Figure 4), except that it was applied for one slice only, and therefore it was faster (less than 1 minute per patient). An averaged lateral ventricles area (LvA) was calculated as the mean of the areas calculated on the 2D sequences.

#### 2.3.3. CSF Flow Measurements

PC MRI was analyzed using a free flow image processing software “Tidam” [13] (http://tidam.fr/) to calculate aqueductal and cervical stroke volumes (ASV and CSV) from CSF flow curves. Stroke volumes represent the volume of CSF displaced through the PC-MRI acquisition plane during a cardiac cycle. They result from the mean of the integration of the positive and negative parts of the CSF curves [13].

### 2.4. Statistical Analysis

Statistical analysis was performed using *R* statistical software (http://www.r-project.org/). 

The distribution of the three groups was evaluated with the Shapiro-Wilk test for normality, to check whether we use parametric or nonparametric tests. A difference was considered significant for a *P* value lower than 0.05.

The two ventricular volume means measured on the BRAVO and coronal 3D and the two area means calculated from the FLAIR T2 and T2*-weighted 2D sequences were compared using Wilcoxon's signed-rank test to verify the reproducibility of volume and area measurements on two different 3D and 2D sequences, respectively. To increase the accuracy of our measurements, averaged volumes measured on the two 3D images and averaged areas measured on the two 2D sequences were calculated to represent the LvV and LvA of each patient, respectively. 

The correlation LvV/LvA was studied using Spearman's correlation test. A linear regression equation was determined to measure an estimation of the lateral ventricles volumes (LvVest) using only the LvA. Correlations between LvV/ASV and LvV/CSV were also studied using Spearman's test.

## 3. Results 

The means of volumes and areas measured on 3D and 2D sequences in our three groups are represented in Table 2. Shapiro-Wilk's normality test revealed that none of our groups followed a normal distribution, and thus we used nonparametric tests for our analysis. 

### 3.1. Lateral Ventricles Volumes

Wilcoxon's test showed that the means of lateral ventricles volumes measures for all patients assessed on the two 3D sequences (T1 Bravo and T1 coronal) were similar (*P* = 0.36). The averaged lateral ventricles volumes (LvVs) we measured (LvV_(VaD)_ = 44 ± 24 mL, LvV_(AD)_ = 51 ± 20 mL, and LvV_(NPH)_ = 180 ± 147 mL) were significantly larger in NPH patients, as expected (*P* = 0.01) (Table 2).

### 3.2. Lateral Ventricles Areas

Means of areas assessed on the two 2D axial images showed no significant differences when compared with Wilcoxon's test (*P* = 0.2). The averaged lateral ventricles areas (LvAs) were also larger in NPH patients (Table 2).

### 3.3. Correlation of LvV with LvA

**Figure 5:**
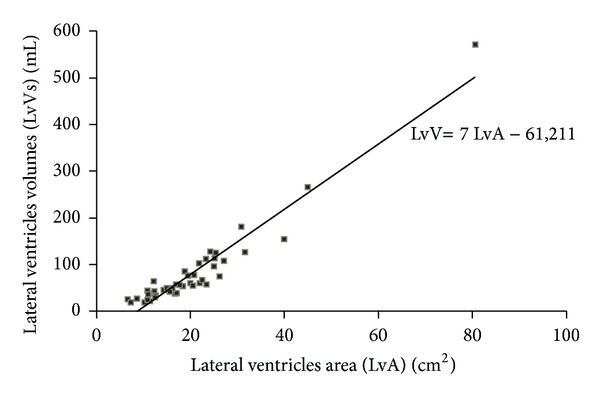
The correlation between LvV and LvA in patients with brain disorders: LvV was positively correlated with LvA (*R* ^2^ = 0.93, *P* < 0.001). Lateral ventricles volumes (LvVs). Lateral ventricle areas (LvAs).

Spearman's regression was computed between LvV and LvA, and a strong positive correlation was detected with a coefficient of determination *R*² = 0.93, *P* < 0.05 (Figure 5). A linear regression equation was determined to represent the LvV for any given LvA using the following equation:

(1) $$\begin{array}{c} {\text{LvV}}_{\left(\text{mL}\right)}=7{\text{LvA}}_{\left(\text{cm}²\right)}-61. \end{array}$$

### 3.4. CSF Stroke Volumes

**Figure 6:**
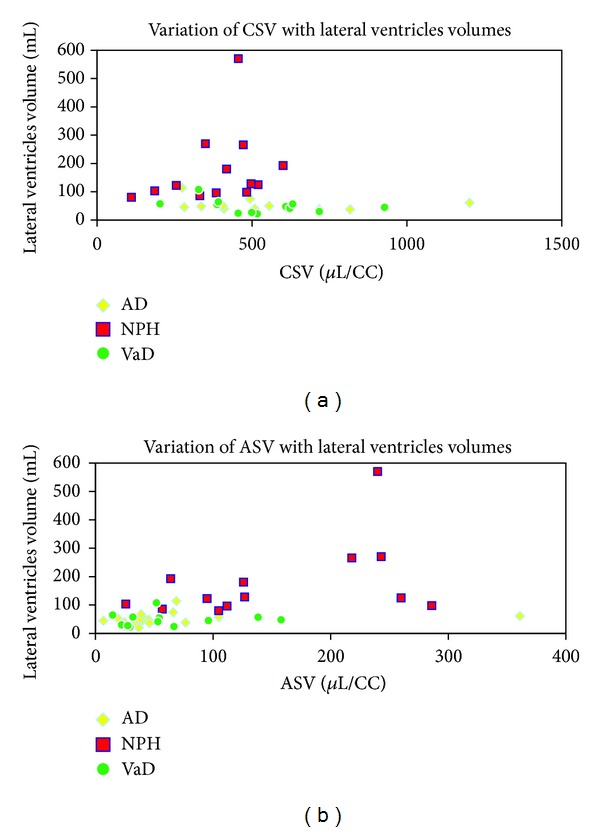
Variation of cerebral (a) and aqueductual (b) stroke volumes (CSV and ASV) with the lateral ventricles volumes in Alzheimer's disease, normal pressure hydrocephaluss and vascular dementia patients. Cerebral stroke volumes (CSVs). Aqueductsal stroke volumes (ASVs). Alzheimer's disease (AD). Normal pressure hydrocephalus (NPH). Vascular dementia (VaD).

The highest ASV and the lowest CSV values were found in NPH patients. On the other hand, no significant differences were observed in ASV or CSV between AD and VaD patients (*P* > 0.1). Moreover, no significant correlations were detected with LvV/ASV or LvV/CSV in any dementia form (Figure 6). ASV and CSV measures in all groups as well as Spearman's correlation results are represented in Table 3. 

### 3.5. Reproducibility

As shown, mean volume and area measurements assessed on two different 3D and 2D sequences, respectively, were significantly similar. The intraobserver variability of LvV and LvA calculations was verified by assessing twice the LvV and LvA for 12 random patients included in our population. Means of LvV and LvA were homogeneous with (LvV = 50 ± 22 mL; LvA = 17 ± 4.46 cm²) for the first measures and (LvV = 50 ± 21 mL; LvA = 17 ± 4.16 cm²) for the second ones. Interobserver variability was verified by asking a group of 20 medicine students to calculate LvV and LvA for 2 patients. Kappa test results showed homogeneous measures for the 2 patients with *κ* = 0.78 for LvV and *κ* = 0.81 for LvA. Our regression model was tested by an experienced clinician by measuring the LvA on the 2D Flair images of 15 patients and then using the equation to estimate the corresponding LvV. Wilcoxon's test between means of the original and estimated LvVs revealed no significant differences (*P* > 0.05). 

## 4. Discussion

In this study, we calculated first the volume of the CSF in the lateral ventricles of patients with AD, NPH, and VaD. The volumes we obtained (particularly LvV_(AD)_ = 51 ± 20 mL and LvV_(VaD)_ = 44 ± 24 mL) were compatible with values found in the literature of LvV assessed by full and semiautomatic segmentation methods in subjects with brain dementia. Barra et al. [14] assessed the LvV using an automatic volumetric measurement with correction of partial volume effects in 5 patients with Alzheimer's disease and found out a total volume of 40 ± 6 mL. Tsunoda et al. [15] evaluated the LvV with a semiautomatic method in patients with NPH and VaD and obtained 76 ± 19 and 46 ± 22 mL, respectively. Also, Nestor et al. [16] studied the ventricular enlargement in 105 AD patients using semiautomatic segmentation software and observed a lateral ventricular volume of 49.9 ± 25.3 mL. 

Estimation of the lateral ventricles volumes is of clinical value for diagnosing brain disorders. The paper demonstrated the feasibility of estimating the volumes from a 2D image. The method depends on the 2D morphological slice that served as a reference and was extracted from common morphological series (T2 FLAIR and T2* WI) covering the whole brain. This 2D slice is obtained when the brain is well centered and the lateral ventricles are normally oriented in terms of rotation around the *x*, *y*, and *z* axes. Images that do not fulfill these conditions must be reformatted or excluded from the population. The accuracy and reproducibility of the slice position are limited by the fact of having only 2 morphological series per patient and 1 series per acquisition, with a slice gap of 6 mm and a slice thickness of 5 mm for both series. However, for all patients, well-positioned images by an experienced technician according to the AC-PC (AC = anterior commissure, PC = posterior commissure) anatomical reference plane were double-checked before the inclusion in the protocol. Moreover, paired comparison of the mean lateral ventricle areas in question measured on the T2-FLAIR and T2* WI 2D series showed no significant differences (*P* = 0.2) in the 3 groups of the population (Table 2). This shows the importance of a well-positioned common reference plane in order to obtain reproducible results.

**Figure 7:**
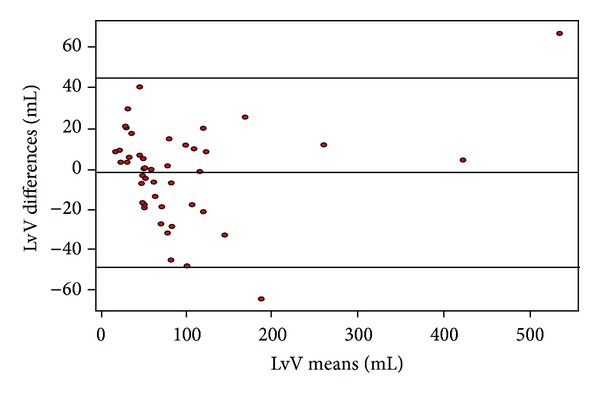
Bland-Altman analysis plot of original and estimated lateral ventricles volumes. The difference between the two measurements lied within the limits of agreement (approximately 95%), and the bias between the two methods was close to zero.

To evaluate the agreement between the LvV estimated from a single 2D section (LvVest) and LvV calculated using semiautomatic segmentation on the 3D images, we used the Bland-Altman analysis. The Bland-Altman plot showed that volumes measured with the two methods lied within the limits of agreement (95%). In addition, the mean difference between the two methods (i.e., bias) was close to zero (Figure 7). This indicates that the two assay methods are systematically producing similar results. Therefore, this method could be acceptable to estimate the LvV from the LvA.

Our CSF flow investigations considered measuring the stroke volume at the aqueductal (ASV) and the C2-C3 cervical level (CSV). Our findings were compatible with values found in the literature with a higher ASV in NPH patients [7]. Besides, we showed that the CSF flow was not correlated with the morphology of the lateral ventricles in any of the three brain disorders. In a previous study, Chiang et al. [8] studied the relationship between the ASV and the ventricular morphology in twenty subjects (10 healthy and 10 patients with NPH) and pointed out a strong correlation of the ASV with the total ventricular and the fourth ventricle volumes (*r* = 0.838 and *r* = 0.76, resp.). The measure of the ventricular size was obtained by a manual tracing of the ventricles boundaries on a 3D T1-weighted scan. These results are in contradiction with our findings proving the absence of any correlation between ASV and LvV with *r* < 0.1, considering that the lateral ventricles represent around 90% of the total ventricular system volume. This contrast could be explained by the fact that the population they studied (mean age = 49 years) was younger (mean age of our population = 73 years), and only 6 out of the 10 NPH patients were diagnosed with suspected NPH, noting that the diagnosis was not clearly described. On the other hand, in accordance with our results, their CSV measurements were not correlated with the ASV. In another study, Bateman et al. [17] found no statistical difference in ASV between patients with AD, VaD, and NPH, with the highest ASV in NPH patients and a large variability within each group, similar to our results. They reported as well that the ventricular size may be correlated with the ASV, noting that the ventricular volume was assessed with a frontal ventricle/cerebral index. They also stated that the magnitude of the ASV depends on the arterial pulse volume, the relative compliance of the arterial tree, in accordance with the tradition of the arterial intracerebral systolic inflow considered as the driving force in the brain [18]. It is true that the driving force in the brain depends mainly on the arterial pulse, but its effects on the CSF as well as the amplitude of the ICP are arteriovenous dependent. An increase of the arterial inflow associated with a simultaneous increase of the venous outflow volume does not affect the ASV, whereas an increase in the resistance venous outflow increases the amplitude of the ICP even if the arterial inflow remains intact. Moreover, the CSF volume in the aqueduct represents only one part of its response to the vascular expansion. The CSF flow in the intracranial subarachnoidal spaces must also be taken into account.

The venous outflow, according to the Monroe-Kellie doctrine, plays a crucial role in modulating the CSF pulsatility. The total intracranial volume change reflecting the blood's inflow/outflow must be considered as an important factor on the ASV as reported in Stoquart-Elsankari et al. [19], showing the preponderant role of the venous system in the regulation of the intracranial pressure and cerebral compliance. On the other hand, Barkhof et al. [20] evaluated the pulsatile CSF flow at the aqueductal level in 11 young (mean age 30 years) and 9 aged controls (mean age 69 years) and found that their phase-contrast aqueductal measurements were not significantly related to the ventricular size or the cortical atrophy. They stated that physiological variables might be important in determining aqueductal CSF pulsatility like the extracerebral and intracerebral CSF volume, the rate of production and absorption of CSF, and the arterial and venous pressure as well. 

The biofluid mechanics may also explain why the ASV cannot be related to the ventricular morphology: in an oscillatory flow as encountered in the aqueduct of Sylvius, the flow is governed by the dimensionless Womersley number Wo [21]: Wo = *d*_*h*_ · (*ρ* · *ω*/*μ*)^1/2^, where *d*_*h*_ is the hydraulic diameter of the duct, *ρ* the density of the CSF, *ω* the cardiac pulsation, and *μ* the CSF viscosity. The morphological parameter that affects this number is therefore the section of the duct represented in our case by the aqueduct of Sylvius. Studies on fluid mechanics have shown that when Wo varies due to an increasing *d*_*h*_, the amplitude of the flow velocity oscillations and therefore the stroke volume act in the same manner [22], which implies that for the same given ventricular volume, the aqueductal stroke volume may vary depending on the aqueductal section. In fact, according to the Hagen-Poiseuille law in standard fluid dynamics notation applied to CSF flow in the aqueduct:

(2) $$\begin{array}{c} Q=\frac{\pi R^{4}}{8\eta }\frac{\Delta P}{L}, \end{array}$$

where *Q* is the volumetric CSF flow rate, Δ*P* is the pressure loss between the 3rd and 4th ventricles, *R* is the radius of the duct, *L* is the length of the duct and *η* is the dynamic fluid viscosity. So according to the equation, the measured CSF flow depends on the radius of the duct and, in consequence, the aqueductal flow section. 

 An interesting normalization of the CSF flow volume would be done by considering the required pressure to displace CSF volume between the 3rd and 4th ventricles, according to the resistance of the aqueduct (defined by *Re* = 8*ηL*/*πR* ^4^). This normalization depends not only on the section plane on the PC MRI but also on a high-resolution anatomical magnetic resonance imaging that permits a precise 3-D anatomical digitalized reconstruction of the entire Sylvius aqueduct. Then, an approximate numerical flow can be computed to measure the flow resistance and thus, calculate the pressure gradient between the 3rd and 4th ventricle [23]. 

If the studied population included subjects with similar ventricular volumes and different Womersley numbers, a significant correlation between ASV and LvV should not be expected. This may explain the differences between our study and the literature and indicates as well that the oscillatory flow parameters must be considered when choosing an appropriate population.

We suggest, in summary, that several intricate factors influence the aqueductal flow during the cardiac cycle: the difference in CSF pressure in the third (V3) and fourth (V4) ventricles, the aqueductal geometry and mainly its section, and the heart rate. This difference is not accessible and represents the difference in infra- and supra-tentorial pressures, or also the importance of the dephasing of the intracranial pressure between these two stages. 

The demonstration of a direct relationship between ventricular volume and the volume of CSF oscillations cannot be established unless the other important parameters (heart rate and section) are constant values in the studied population.

## 5. Conclusion

Our study yielded an estimation of the ventricular volume with respect to published data and a relevant management of partial volume effects. It showed also that the LvV is significantly proportional to the LvA of the given 2D reference section and therefore, it could be used for the longitudinal follow-up of patients with brain disorders even if 3D MR images are unavailable. Furthermore, results demonstrated the nonexistence of any particular correlation between the lateral ventricles volume and the CSF pulsatilities at the aqueductal and cervical levels in AD, NPH, or VaD. The CSF circulation seems to be ruled by a highly complex system and may depend on many physiological parameters other than the ventricular morphology. 

## Figures and Tables

**Table 1:** Detailed parameters of MRI and PC-MRI scans applied to all patients.

	2D T2 FLAIR	2D axial T2*	2D T1 Sagittal	3D T1 coronal	3D T1 BRAVO	2D PC MRI
FOV (cm^2^)	24 × 24	24 × 18	26 × 26	22 × 19.8	25 × 23	14 × 14
Matrix	384 × 224	320 × 256	416 × 256	512 × 256	256 × 256	256 × 128
Slice Th (mm)	5	5	5	2	1.4	5
TE (ms)	152	13	8.4	4.6	4	7
TR (ms)	9002	620	220	13.1	10	15
Nex	1	1	1	0.5	0.5	1

Field of view (FOV). Slice thickness (Slice Th). Number of excitations (Nex).

**Table 2:** Mean values of lateral ventricles volumes and lateral ventricles areas measured in AD, VaD, and NPH patients.

Population	Lateral ventricles volumes (mL)			Lateral ventricles areas (cm²)		
	3D sequences		Mean (LvV)	2D sequences		Mean (LvA)
	T1 BRAVO	T1 coronal		Axial T2 Flair	Axial T2*	
AD (Mean ± SD)	52 ± 12.2	51 ± 21.9	51 ± 20	16 ± 5.8	16 ± 6.3	16 ± 6
VaD (Mean ± SD)	43 ± 24	46 ± 25.1	44 ± 24	16 ± 5.5	16 ± 5	16 ± 5
NPH (Mean ± SD)	182 ± 149	178 ± 146	180 ± 147	33 ± 19	35 ± 20	34 ± 18

Standard Deviation (SD); Alzheimer's disease (AD); Vascular dementia (VaD); Normal pressure hydrocephalus (NPH).

**Table 3:** Variations ASV and CSV according to the brain disorders and its correlation with the corresponding lateral ventricles volumes using Spearman's test.

	AD	NPH	VaD
ASV (mean ± SD *μ*l/CC)	76 ± 60	108 ± 72	62 ± 46
CSV (mean ± SD *μ*l/CC)	551 ± 264	417 ± 222	524 ± 193
Correlation (Asv/LvV)	*R* ^²^ = 0.06; *P* = 0.073	*R* ^²^ = 0.0026; *P* = 0.5	*R* ^²^ = 0.0079; *P* = 0.88
Correlation (Csv/LvV)	*R* ^²^ = 0.05; *P* = 0.23	*R* ^²^ = 0.006; *P* = 0.23	*R* ^²^ = 0.15; *P* = 0.15

Aqueductal stroke volumes (ASVs). Cervical stroke volumes (CSVs).
